# Identifying Child Abuse in Patients With Femur Fractures Through Pathway Compliance: A Pilot Study

**DOI:** 10.7759/cureus.72044

**Published:** 2024-10-21

**Authors:** Kylie Scallon, Shirley Wiggins, Kaeli K Samson, Adil Shah

**Affiliations:** 1 Trauma, Children's Nebraska, Omaha, USA; 2 Education, Children's Nebraska, Omaha, USA; 3 Biostatistics, University of Nebraska Medical Center, Omaha, USA; 4 Pediatric Surgery, Children's Nebraska, Omaha, USA

**Keywords:** clinical pathway, femur fracture, non-accidental trauma, orthopedics, pediatrics

## Abstract

Background

Child abuse is a significant cause of morbidity and mortality in children. A thorough history and physical exam is critical to identifying abuse. Standardized screening tools as well as clinical pathways can assist with identifying patients who may have an injury secondary to abuse. There are varied recommendations for the upper age in which to routinely evaluate pediatric patients for child abuse.

Objectives

The primary objective of this study was to describe the outcomes of the implementation of an evidence-based clinical pathway for suspected non-accidental trauma for all pediatric patients aged five years and under presenting with a femur fracture. This pathway includes orders for imaging, labs, and consults. In addition, the characteristics of this population and the findings were described.

Methods

A retrospective study of patients at a single institution who met the National Trauma Data Standard registry inclusion criteria and had a femur fracture was performed. Variables analyzed included age, demographics, mechanism and location of injury, admission status and service, injury severity, hospital characteristics, and discharge status. Patients with a metabolic bone disease were excluded.

Results

There were 200 patients who met the inclusion criteria. Thirty-two patients had a diagnosis of confirmed or suspected child abuse. While all 32 patients had a skeletal survey performed, only 23 (71.9%) had the complete workup per the clinical pathway, and 21 (65.6%) had a diagnosis of confirmed child abuse.

Conclusion

Clinical pathways are established to provide the standardization of clinical assessments and interventions; however, this process relies on a single individual to make a judgment determining whether or not to implement the pathway. Children presenting to an emergency department with confirmed or suspected child abuse are a vulnerable population. A child abuse diagnosis is a clinical judgment; however, clinical pathways aid in the diagnosis in hopes to stop any further abuse. For a pathway to be successful, each step needs to be addressed.

## Introduction

In 2022, there were over 550,000 children victims of maltreatment [1]. Of this number, an estimated 1990 children died from abuse. Systematic screening of all high-risk pediatric emergency department (ED) admissions can increase the detection of child abuse [2]. Non-accidental trauma (NAT) clinical pathways (CPWs) have been described as an intervention to increase the identification of child maltreatment, ensure children receive proper follow-up with a child abuse pediatrician, and reduce healthcare bias [3]. Interventions within a CPW establish safe, quality care outcomes for patient-care management that directs care based on standards established in evidence and research. These interventions can be implemented by a multidisciplinary team for specific populations and diagnoses. These CPWs often start by targeting a high-risk population, such as patients presenting with femur fractures, for the NAT detection CPWs.

It is important to screen for NAT in order to provide appropriate care and follow-up for the child and the family and to prevent future trauma [1]. Providers and the multidisciplinary teams examining a young child who presents with a femur fracture must have a heightened awareness of the potential of NAT. The American Academy of Orthopaedic Surgeons (AAOS) strongly recommends that children younger than 36 months with a diaphyseal femur fracture be evaluated for NAT [4]. It is important to note that the absence of other injuries does not exclude NAT as an etiology [5]. Failing to identify the risk factors and therefore missing NAT in a child such as those with femur fractures hold the possibility of severe consequences [6]. Standardization of practice such as implementing a CPW is a valuable means of improving the management of patient outcomes and care [7-9]. This study aims to identify vulnerability characteristics that predisposed patients to NAT with the application of a standardized CPW.

The primary objective of this study was to describe the outcomes of the implementation of an evidence-based CPW for suspected NAT for all pediatric patients aged five years and under presenting to our hospital with a femur fracture. In addition, the characteristics of this population and the findings were described.

## Materials and methods

Study design

This is a single-center, retrospective cohort study, evaluating compliance with a CPW. Following IRB review by the University of Nebraska Medical Center, it was determined that this did not constitute human subject research as defined at 45CFR46.102; therefore, no further IRB application was required.

Population and setting

Our tertiary pediatric hospital is a free-standing facility, as well as an American College of Surgeons verified level II pediatric trauma center with an annual volume of 38,000 patients. Located in the Midwest, it is the only pediatric hospital in the state. There are 112 intensive care unit beds, and the hospital is licensed for 225 inpatient beds. There are approximately 800 patients per year added to the trauma registry.

**Figure 1:**
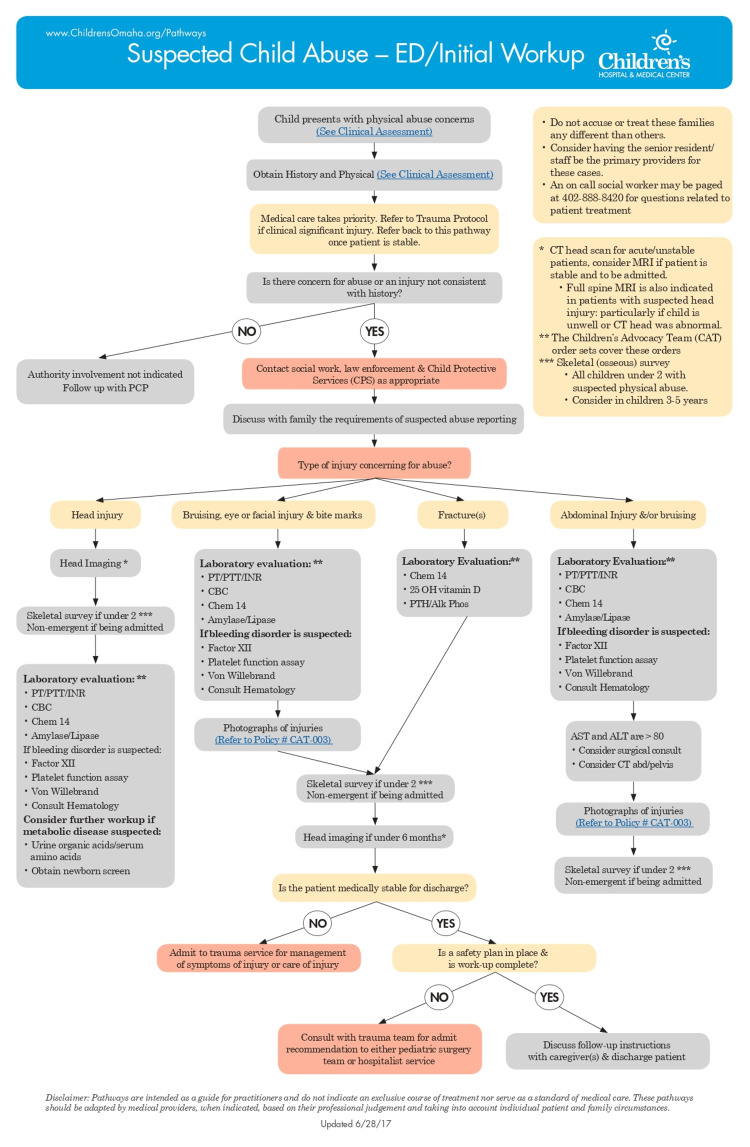
Emergency Department Physical Abuse Pathway Reference: [10] ED: emergency department; CAT: Children's Advocacy Team; PCP: primary care provider; CPS: Child Protective Services; PT: prothrombin time; PTT: partial thromboplastin time; INR: international normalized ratio; CBC: complete blood count; AST: aspartate transaminase; ALT: alanine transaminase

In June 2017, the Emergency Department Physical Abuse Pathway was implemented (Figure 1).

This pathway was developed primarily by our hospital child protection team to assist with the care of children with suspected NAT. Using evidence-based information, a team including members from emergency medicine, social work, and case management met with our child protection team to determine appropriate steps and workflows. The pathway is in place to ensure patients with a potential NAT diagnosis receive the same standardized workup and subspecialty consultations, thus decreasing the opportunity for bias. The pathway is accessible on the internal network of the hospital and is associated with standardized order sets within the electronic health record (EHR). The pathway is also available externally. Orders include imaging, labs, and consults with social work and child abuse pediatrician. In patients presenting with suspected NAT, the CPW indicates laboratory evaluation to screen for a metabolic disorder followed by further evaluation depending on age. For those less than two years of age, a skeletal survey should always be obtained, and for those under six months of age, head imaging should be obtained. A skeletal survey is to be considered but is not required in patients two to five years old.

We performed this program evaluation to identify compliance using our trauma center's suspected child abuse CPW. Our CPW recommends age five as the oldest child considered for a skeletal survey; therefore, we used age five as our upper age limit for data collection. We performed a retrospective review of data from 2017 to 2022 of patients who presented at our institution with a femur fracture and who met the National Trauma Data Standard inclusion criteria and were captured in the trauma registry.

Data collection

The trauma registry database was searched to include all pediatric patients admitted from June 2017 through December 2022. Inclusionary data was children from birth through five years of age and the International Classification of Diseases, Tenth Revision (ICD-10), diagnosis of a femur shaft fracture, including proximal, mid-diaphyseal, and distal fractures. To be captured in the trauma registry, the patient must have been admitted to a level II trauma center or transferred from an outside hospital. A report was created from the trauma registry. Data obtained from the registry report included age, gender, and race of the patient, imaging obtained, consulting services, injury side, type of fracture, mechanism of injury, comorbidities present, primary insurance, ED disposition, and, if admitted to the hospital, admitting service and hospital disposition. The type of fracture was determined by the radiology report and the orthopedic note. Suspected NAT was defined as the patient receiving any part of the CPW, which includes a skeletal survey, labs, or head CT if under six months old. If those studies were negative and the ED provider determined the patient's injury fit the explanation, the mechanism of injury was not labeled as assault and was classified according to the injury explanation. Confirmed NAT was defined as a confession from a caregiver or the child protection team note stating that there was no explanation for injuries or that injuries were suspicious for abuse. Patients over five years of age and those with known metabolic bone diseases and pathologic fractures were excluded.

Statistical analysis

Descriptive statistics for continuous data are given as medians and interquartile ranges ((IQRs) representing the range of the middle 50% of the data). Differences in the age of children between subgroups within fracture characteristic variables of interest (e.g., type, location, side) were assessed using the Kruskal-Wallis tests; only subgroups which had counts of at least 3 were included in the analysis. Significant Kruskal-Wallis tests were followed up with post hoc pairwise comparisons of subgroups using the Wilcoxon rank-sum tests, and the associated p-values were Bonferroni-adjusted based on the number of analyzed subgroups within a given fracture characteristic variable. All analyses were performed using SAS software version 9.4 (SAS Institute Inc., Cary, NC, USA).

## Results

**Figure 2:**
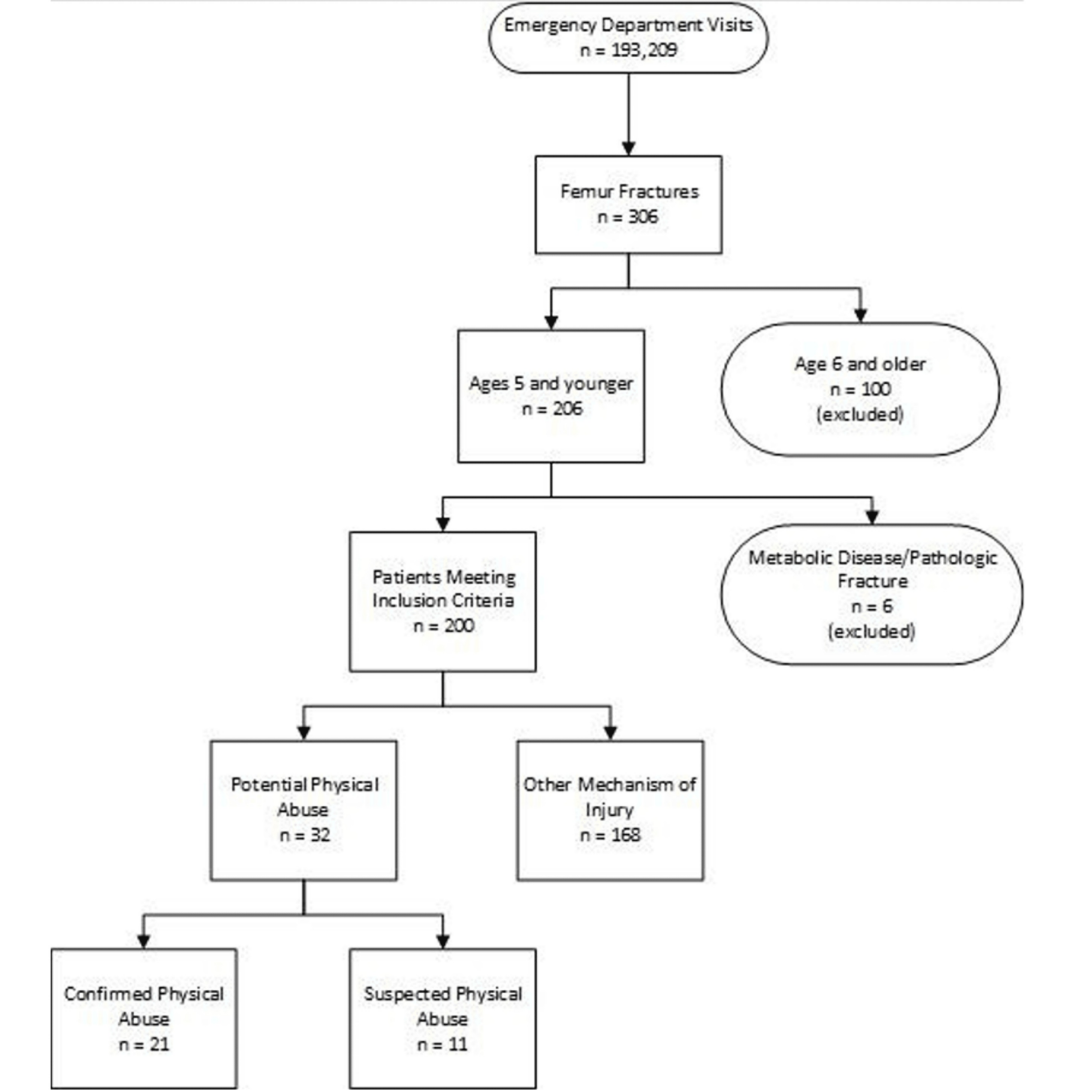
Patients meeting the inclusion criteria

There were 206 patients who were identified as having a femur fracture; however, six did not meet the inclusion criteria due to a metabolic disorder or pathologic fracture and were removed from the results, leaving a total population of 200 (Figure 2).

The majority of included patients were Caucasian, male, and two years old (Table 1).

**Table 1:** Demographic data of patients Race was defined by the parent or caregiver; other race indicates parent or caregiver identified the patient as something other than the listed races

	All patients (n=200) n (%)
Age in years	
<1	37 (18.5%)
1	32 (16%)
2	61 (30.5%)
3	35 (17.5%)
4	23 (11.5%)
5	12 (6%)
Gender	
Female	64 (32%)
Male	136 (68%)
Race	
American Indian	1 (0.5%)
Asian	1 (0.5%)
Black or African American	18 (9%)
Caucasian	161 (80.5%)
Two or more races	1 (0.5%)
Other race	18 (9%)
Primary insurance	
Private	91 (45.5%)
Medicaid	98 (49%)
Military (Tricare)	5 (2.5%)
Self-pay	6 (3%)

**Figure 3:**
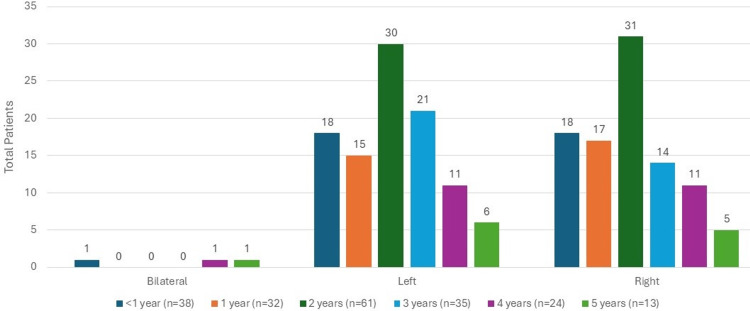
Fracture side

Descriptive variables for this population identified that 101 (50.5%) fractures were on the left side, 96 (48%) fractures were on the right side, and three (1.5%) patients had bilateral femur fractures (Figure 3).

**Figure 4:**
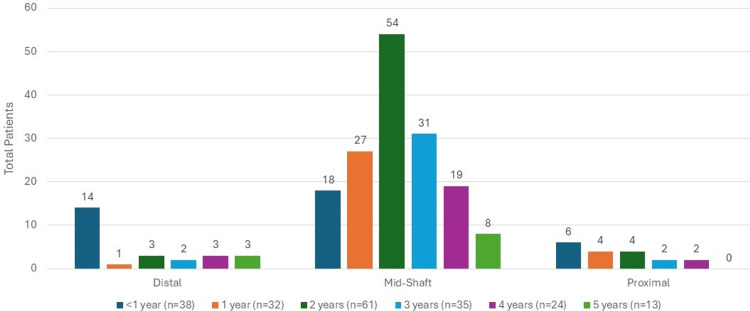
Femur fracture location

Thirty-four (17%) of the patients had additional injuries. The locations of 203 femur fractures were mid-shaft (157, 77.3%), distal (26, 12.8%), or proximal (20, 9.9%) (Figure 4).

**Figure 5:**
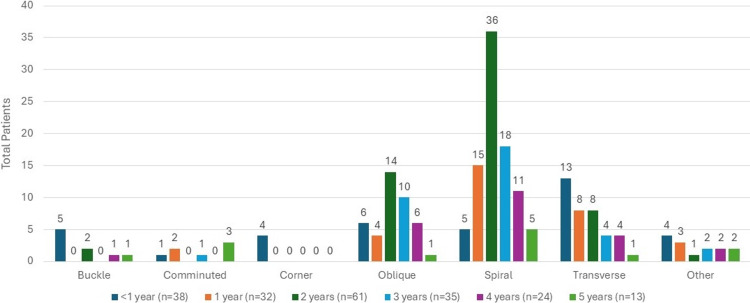
Fracture type

Morphologic features of all fractured femurs indicated 90 (44.3%) fractures were spiral fractures, 41 (20.2%) were oblique fractures, 38 (18.7%) were transverse fractures, nine (4.4%) were buckle fractures, seven (3.4%) were comminuted fractures, four (2%) were corner fractures, also known as classic metaphyseal lesions, and 14 (6.9%) were other proximal or distal fractures (Figure 5).

**Figure 6:**
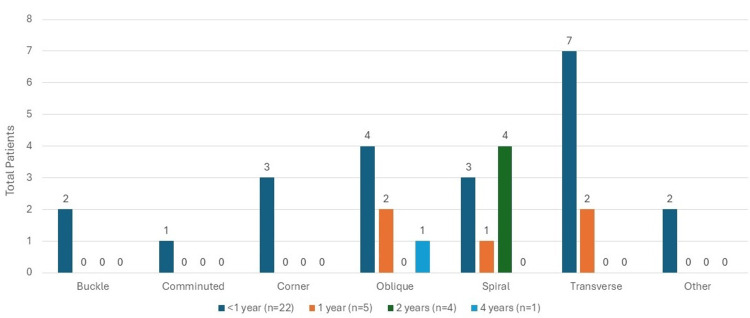
Fracture type in confirmed or suspected abuse

In looking specifically at NAT patients, the top three fracture types showed nine (28.1%) fractures were transverse, eight (25%) were spiral, and seven (17.9%) were oblique (Figure 6).

The distribution of age was assessed between subgroups of the fracture characteristic variables of interest, specifically for subgroups with counts of at least three children; however, all subgroups were visualized using age groups in the associated figures. We found that there was a significant association between age and fracture type (p=0.001), as can be seen in Figure 5, where children with spiral fractures tended to be older (median age=2 years (IQR: 2, 3)) than children who had transverse fractures (median age=1 (IQR: 0.7, 2; p=0.01)). In addition, there was a significant association between age and fracture location (p=0.01), as can be seen in Figure 4, where children with shaft fractures tended to be older (median age=2 years (IQR: 1, 3)) than children with distal fractures (median age=0.6 (IQR: 0.3, 3; p=0.02)). Significant associations were not detected between age and fracture side in the whole study sample (p=0.58), as shown in Figure 3, nor between age and fracture type when only looking at children with suspected or confirmed physical abuse (p=0.14), as can be seen in Figure 6.

The top three categories for the mechanism of injury were a fall in 125 (62.5%) patients, assault in 21 (10.5%) patients, and other blunt mechanism in 40 (20%) patients (Table 2).

**Table 2:** Mechanism of injury category MVC: motor vehicle collision

	All patients (n=200) n (%)
Assault	21 (10.5%)
Bicycle	4 (2%)
Fall 1-6 m	22 (11%)
Fall under 1 m	103 (51.5%)
MVC	6 (3%)
Other blunt mechanism	40 (20%)
Pedestrian	4 (2%)

The definition of other blunt mechanism included accidental hits, strikes, or twists by another person, crushing injury, being caught between moving or stationary objects, and striking against objects. At least one comorbidity was identified in 17 (8.5%) of the patients. The payor mix of the patient population showed 98 (49%) patients had Medicaid, 91 (45.5%) patients had private insurance, six (3%) patients were self-pay, and five (2.5%) patients had military (Tricare) insurance.

Orthopedic surgery was the admitting provider for 159 (79.5%) of the admitted patients (Table 3).

**Table 3:** Admission, treatment, and discharge of patients

	All patients (n=200) n (%)
Initial admission location	
Floor	41 (20.5%)
Routine/discharge	10 (5%)
Intensive care unit	4 (2%)
Observation unit	92 (46%)
Operating room	53 (26.5%)
Admitting team	
Critical care	1 (0.5%)
Hospitalist	6 (3%)
Orthopedics	159 (79.5%)
Trauma	24 (12%)
Other	10 (5%)
Day of the week admission	
Sunday	25 (12.5%)
Monday	25 (12.5%)
Tuesday	23 (11.5%)
Wednesday	26 (13%)
Thursday	26 (13%)
Friday	30 (15%)
Saturday	45 (22.5%)
Discharged to	
Foster care	13 (6.5%)
Home	184 (92%)
Rehabilitation setting	3 (1.5%)

Of the admitted patients, 184 (92%) were discharged to home, 13 (6.5%) were discharged to foster care, and three (1.5%) were discharged to a rehabilitation facility.

Of the total population who should have had CPW applied to their care, we further explored the data on those identified as confirmed and suspected NAT (Table 4).

**Table 4:** Completed assessment in confirmed or suspected abuse CPW: clinical pathways

	All patients with completed assessment (n=32) n (%)
Mechanism of injury	
Child physical abuse confirmed	21 (65.6%)
Child physical abuse suspected	11 (34.4%)
CPW required assessments completed	
Skeletal survey	32 (100%)
Skeletal survey <2 years of age	27 (100%)
Laboratory drawn	23 (71.9%)
Head CT	17 (53.1%)
Head CT child <6 months	13 (86.7%)
Psychosocial follow-up completed	
Social work consultation	31 (96.9%)
Child protection team consultation	25 (78.1%)

All 32 (100%) patients in this sample received a skeletal survey. Of the 21 patients with confirmed abuse, eight (38%) of the skeletal surveys were positive for additional injuries, including long bone fractures, rib fractures, skull fractures, spinal fractures, and facial fractures. There was one instance in a suspected abuse case where social work was not consulted. The ED attending did not assess a need to consult social work, as the skeletal survey completed on this patient was negative for any additional fractures and the mechanism of injury described fit the description of the injury. The overall review of our pathway compliance demonstrated that 23 (71.9%) patients had the complete workup per the CPW.

## Discussion

NAT in a child can be a diagnostic challenge, largely due to the variable clinical presentation. Children may arrive at the ED with a parent or care provider, be referred to the ED by the primary care provider, or be transferred from an outside hospital either to the ED or as a direct admission to the hospital. It is crucial to have a high index of suspicion for NAT when children present to healthcare facilities with a fracture [11].

Healthcare providers must maintain constant awareness that NAT is in the differential diagnosis for a long bone fracture, especially in non-ambulatory children. It is critical to provide a complete assessment when the history is not consistent with the pattern of injury [12]. Improper recognition of child abuse at the child's initial presentation can lead to significant and subsequent morbidity [13].

It was previously thought that spiral fractures were reported to be the most common feature of diaphyseal long bone fracture; however, more recently, transverse fractures were recognized to be the most common diaphyseal patterns [14]. Our data supports this, as shown in Figure 6. There are no fractures pathognomonic for child abuse, and therefore, a detailed history and physical examination is imperative so the proper workup and consults can be performed [14]. 

With the Institute of Medicine's call to improve the quality and safety of patient care, a multitude of interventions (care maps, algorithms, etc.) have been introduced [15]. An early study examined the definitions and names used for standard care interventions in order to provide an operational definition of the term CPW as a method of managing patient care for a specifically defined population during a predetermined period [16]. They acknowledged that the key elements of a CPW should include evidence-based guidelines, best practices, communication, coordination of roles, and sequencing of the activities of a multidisciplinary team [16]. This process ensures that outcomes are monitored and documented and variances are measured. CPWs have been shown to decrease medical expenses and length of stay, promote multidisciplinary collaboration, and improve patient outcomes [8]. CPWs help to ensure a systematic approach is taken to each patient and helps to eliminate bias.

CPWs continue to be developed for specific populations to improve care processes and maximize positive patient outcomes through efficient use of healthcare resources [8]. Several studies have found that CPWs not only improve patient care but also reduce variability in outcomes [15]. Application of a NAT CPW provides specific evidence-based guidelines to ensure compliance with key assessments such as the application of skeletal surveys in child abuse screening [17]. CPWs focused on NAT have been implemented with the goal of identifying cases concerning child abuse and ensuring the child and family receive the appropriate workup and assessment [3]. The presence of a CPW augments providers to identify this diagnosis.

Based on our findings, our team identified compliance with the CPW was dependent on the assessment of the ED attending; unfortunately, the compliance rate was 71.9%. The purpose of the CPW is to standardize the workup. However, this process relies on a single individual to make a judgment determining whether or not to implement the pathway. A diagnosis of injury is best made by comparing the history provided with the child's injuries. As previously stated, the AAOS recommends further investigation in patients younger than 36 months presenting with femur fractures. The Society for Pediatric Radiology recommends skeletal surveys in children over 24 months in situations where children are either non-verbal or developmentally delayed and therefore unable to localize injuries [18]. Our institution had one four-year-old patient diagnosed with NAT by utilizing the CPW. Our CPW recommends a skeletal survey up to age five if potential concerns; the verbiage in the CPW assisted in identifying in NAT. This situation would not have been identified if screening for NAT was halted at 36 months.

The use of EHR for the consistent application of CPW guidelines is facilitated by the application of electronic triggers to inform decision-making that ensures the completion of both evidence-based assessments and testing while also preventing racial and socioeconomic bias in the care of this population [17,19]. The integration of a CPW for NAT into the decision support system of an EHR promotes the adherence of providers and the multidisciplinary team with the American Academy of Pediatrics evidence-based guidelines for physical abuse screening [19]. Our institution is in the process of implementing an EHR screening tool. The addition of this tool along with our already existing CPW will continue to help eliminate bias and ensure this vulnerable population is identified and appropriately treated.

Limitations

As the trauma registry data are manually entered, there is the potential to have had information entered incorrectly; therefore, a patient may have been unintentionally excluded in the retrospective search. The patients are identified by reviewing a daily log; with this, it is possible a patient may have been missed during this review. Another significant limitation of the study is the lack of data for comparison prior to the CPW being instituted. The trauma registry was not fully utilized in patient data collection until 2017, and we were unable to access all necessary data points needed for a comparison.

## Conclusions

This study identified an inconsistency in completing the entire CPW. With this insight, the team's next steps involve implementing an EHR screening tool; this will give an added layer of confidence in identifying potential NAT patients and ensuring they get properly evaluated with the CPW. Since the completion of the data collection for this study, our team updated the CPW in May 2024 to include criteria to admit all NAT patients to pediatric trauma surgery. The expectation is for the pediatric trauma surgery team to manage all NAT patients, as this population requires multiple consults due to a clear potential for polytrauma. With these interventions, our overall goal remains to provide care for all NAT patients under a single specialty service that coordinates care with all consulting care teams to ensure CPW compliance for all suspected NAT cases. We recommend other programs consider utilizing a CPW in its entirety to guide their providers in treating NAT patients. 
